# Prediction of infection risk in rheumatoid arthritis patients treated with biologics: are we any closer to risk stratification?

**DOI:** 10.1097/BOR.0000000000000598

**Published:** 2019-03-01

**Authors:** Meghna Jani, Anne Barton, Kimme Hyrich

**Affiliations:** a Arthritis Research UK Centre for Epidemiology, Centre for Musculoskeletal Research, The University of Manchester; b Arthritis Research UK Centre for Genetics and Genomics, Division of Musculoskeletal and Dermatological Sciences, The University of Manchester; c NIHR Manchester Biomedical Research Centre, Manchester Academic Health Science Centre, Manchester University NHS Foundation Trust, Manchester; d Rheumatology Department, Salford Royal NHS Foundation Trust, Salford, UK

**Keywords:** anti-tumour necrosis factor, biologics, infection, rheumatoid arthritis, stratification

## Abstract

**Purpose of review:**

There are currently several available biologics for rheumatoid arthritis (RA) with similar efficacy in most trials. A major consideration therefore in choosing a biologic, continues to be safety concerns such as infection. Considerable advances have been made in the understanding of biologic safety on a population level; however, how close are we to stratifying risk for individual patients? This review discusses evidence published in the last year, with reference to key previous literature.

**Recent findings:**

Comparative safety of biologics has been studied in observational cohorts, with a possible increased risk of serious infection in tocilizumab-treated patients compared with etanercept. Rheumatoid arthritis patients on biologics are often on concomitant medications such as steroids and opioids, and the advances in relation to infection are summarized. Pharmacological biomarkers and optimizing existing risk prediction scores may allow better future risk stratification.

**Summary:**

Improved quantification of personalized benefit:harms would allow better-informed decisions, reduction of infection-associated morbidity as well as direct/indirect costs associated with biologics. Although advances have been made to better understand and predict risk, future studies are likely to require a range of novel data sources and methodologies for the goal of precision medicine to be truly realized.

## INTRODUCTION

Biologics such as tumour necrosis factor inhibitors (TNFis) have transformed the treatment of chronic inflammatory conditions such as rheumatoid arthritis (RA). A rapidly evolving armamentarium of biologic therapies that now includes biosimilars, excitingly provides more choice and therapeutic options for RA patients than ever before. However, with most biologics reported to have similar efficacy in RA, clinicians are often required to tailor treatment decisions based on risk of adverse events for the individual patient. The risk of infection is one of the most important considerations before starting biologic agents, as it represents a substantial source of morbidity and mortality in RA patients [1]. Therefore, the aim of this review is to summarize the latest evidence to inform stratification of patients based on infection risk and includes information on patient characteristics, choice of biologic, concomitant therapy, biomarkers and the utility of available risk prediction scores for infection. 

**Box 1:**
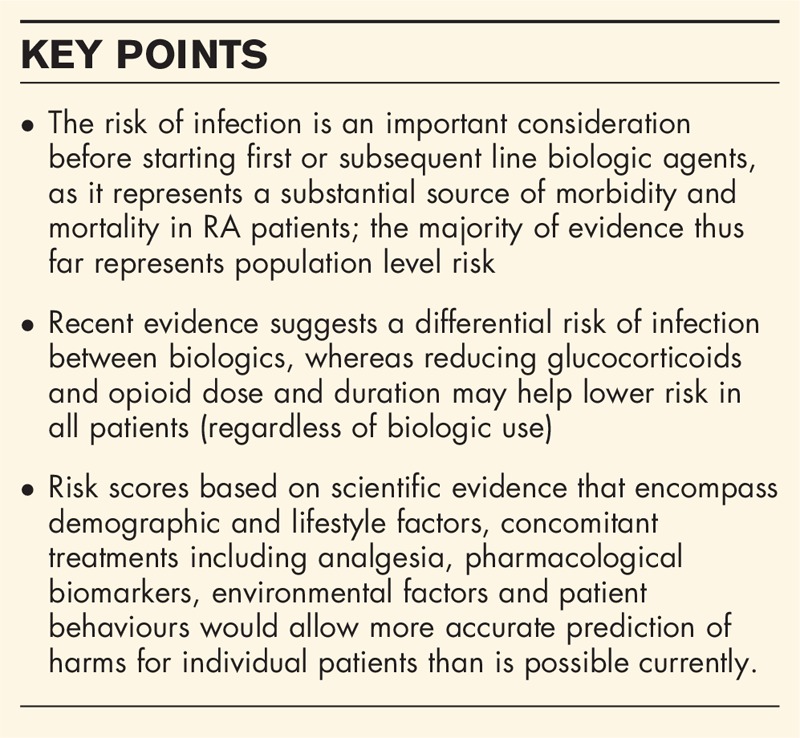
no caption available

## BURDEN OF INFECTION IN RHEUMATOID ARTHRITIS-TREATED BIOLOGIC PATIENTS

The increased risk of infection seen in RA is complex and likely multifactorial. High disease activity, multimorbidity, treatment/disease-related immunosuppression and polypharmacy all likely contribute. Serious infections are defined as events resulting in hospitalization or death. A UK-based real-world study that surveyed RA patients reported 8% required hospitalization because of serious infection each year [2]; however, hospitalized infections are likely to be an underrepresentation when considering the overall burden of infections. A recent cross-sectional study in RA patients reported a tripling of sepsis rates between 1993 and 2013 in the United States (1.9–6.4%) [3], consistent with other sepsis studies in the US general population [4]. This may be attributed to an aging population with more comorbidities, increased use of immunosuppressant drugs, spread of multiresistant pathogens or to better International Classification of Diseases-9 coding of sepsis over time.

TNFi therapies are known to be associated with an increased serious infection risk in comparison to conventional synthetic disease-modifying antirheumatic drugs (csDMARDs), with a time varying risk highest in the first 6–12 months of treatment [5,6]. One of the largest meta-analyses to date, reported a 31% increased risk of serious infections in standard dose biologic-treated RA patients compared to csDMARDs {odds ratio (OR) 1.31 [95% confidence interval (CI): 1.09, 1.58} [7]. In the clinical trial population studied, the absolute increase in number of serious infections associated with biologics was reported as six per 1000 patients treated per year for standard dose biologics. Although such reporting of absolute risk promotes informed decision-making, population estimates can be difficult to extrapolate to the individual patient. Clinicians and patients ideally need a better understanding of differences in infection risk between biologics, the influence of patient characteristics and concomitant treatments to allow better stratification of infection risk and more personalized treatment choices.

## STRATIFICATION OF INFECTION RISK

### Patient demographics

A number of patient factors have been evaluated in association with infection risk. Consistently, increasing age [8] and comorbidities [9] have been associated with both serious infection and opportunistic infections. In a recent retrospective single-centre study evaluating patients aged more than 65 years commencing a biologic, the most common adverse event leading to treatment discontinuation was infection [10]. Previous studies have demonstrated an increased risk of infection in patients with comorbidities such as chronic obstructive airways disease, interstitial lung disease and chronic renal failure [11,12]. The accrual of comorbidities resulting in multimorbidity with consequential polypharmacy is likely to predispose such patients to a higher infection risk. Therefore, strategies to minimize infection at the outset in these patients such as advice about vaccinations and thorough education about specific risk behaviours that increase risk of infection should be carefully exercised.

## COMPARATIVE RISK OF INFECTION BETWEEN BIOLOGICS

### Types of infection

The most common types of serious infections overall in biologic-treated RA patients are respiratory infections [22/1000 patient-years (pyrs)], skin and soft tissue (11/1000 pyrs), genitourinary (6.2/1000 pyrs) and bone/joint infections (5.4/1000 pyrs) [13^▪▪^], although few studies have suggested the rates of these infections vary significantly between biologic therapies. Thirty-day mortality following serious infection remains is also high, with mortality rate of 10.4% (95% CI 9.2, 11.6%) observed within the British Society for Rheumatology Biologics Register for RA (BSRBR-RA). Sepsis/bacteraemia was associated with the highest 30-day mortality at 45% (95% CI 33%, 61%) [13^▪▪^].

### Between tumour necrosis factor inhibitor drugs

An infection type that appears to have a clear differential risk across available TNFi therapies is tuberculosis (TB). The rate of TB has fallen with the introduction of prescreening precautions [9], yet the continued risk of TB reactivation in TNFi-treated patients requires ongoing vigilance and investigation in any patient with symptoms suggestive of active TB regardless of their prebiologic screening results. The risk of TB reactivation appears lower in etanercept-treated patients compared with the monoclonal antibodies such as infliximab and adalimumab [14], as observed in several studies. In patients with risk factors for TB, etanercept may be the TNFi drug of choice. Risk factors may include a history of contact to a case of active TB, being born or extended living (>3 months) in TB prevalent regions (crude incidence ≥20/100 000 per year), history of living or working in prisons, homeless shelters, healthcare facilities providing care to TB patients or in patients with a history of intravenous drug use [15].

### Tumour necrosis factor inhibitors versus non-tumour necrosis factor inhibitor biologics

For the most common types of serious infections, the majority of studies do not conclude a clinically meaningful difference between classes of drugs, after adjustment for baseline differences between patients. However, interpreting the evidence with available data sources can be challenging. There are scarce head to head randomized controlled trials between biologics. The primary outcome of the few that exist focus on efficacy and lack power or long-term follow-up to detect significant differences in rare events such as serious infection. A previous large meta-analysis of biologics in RA reported a significantly higher rate of serious infection with anakinra and certolizumab pegol compared with a control population [16]. However, network meta-analysis compare heterogeneous groups of patients with differences in recruitment year, duration of disease, follow-up duration and covariates and rely on indirect comparisons between drugs that may be prone to error.

### Rituximab

More recent observational studies have started to explore the comparative safety of biologics within and across class. As these data reflect real-world clinical decisions and patients are not randomly assigned to medication, such studies may be prone to channelling bias and results have been conflicting. Results from the US Brigham and Women's Hospital Rheumatoid Arthritis Sequential Study (BRASS) registry suggest no significant differences in infection risk between TNFi and non-TNFi-treated patients [17]. Conversely, a recent study from BSRBR-RA reported a differential risk between biologics. The unadjusted results suggested a higher incidence of infection in rituximab-treated patients than etanercept; however, after adjustment using baseline characteristics, the differences were no longer significant [13^▪▪^]. Patients who receive rituximab in the United Kingdom, receive it second/third line and are older, with longer disease duration, more comorbidities and on multiple other medications. A sensitivity analysis which looked at subsequent line biologics only also found no difference between rituximab and etanercept, consistent with an earlier report from the same dataset restricted to second-line biologic therapy [18].

### Tocilizumab and other biologics

The BSRBR-RA study also reported a statistically significant increased risk of serious infection with tocilizumab-treated RA patients compared with etanercept [hazard ratio (HR) 1.21 (95% CI 1.01, 1.79)] [13^▪▪^]. This may reflect unmeasured confounding as tocilizumab patients may have failed prior therapies and may be inherently different; however, the results remained significant when the analysis excluded biologic-naïve patients. In terms of efficacy, the tocilizumab monotherapy versus adalimumab monotherapy for treatment of rheumatoid arthritis (ADACTA) trial found that tocilizumab monotherapy had superior efficacy compared with adalimumab [19]. Therefore, it is a good example where the balance between efficacy and safety needs to be tailored to the individual patient requiring monotherapy depending on individual characteristics, followed by informed decision-making between the clinician and patient. An improved efficacy profile with tocilizumab monotherapy compared to a TNFi (ΔDAS28 difference at 24 weeks of −1·5, 95% CI −1·8, −1·1) balanced against a possible increased SI risk of 21% with tocilizumab compared to TNFis such as etanercept.

In the same UK observational study, the rate of serious infection for certolizumab was lower than etanercept, in contrast to a previous meta-analysis [16], but the results were not replicated in several sensitivity analyses suggesting residual confounding. As certolizumab was licensed later than other TNFis, there may be inherent differences in patients recruited to the study on the drug that may not be captured by covariate adjustment [20].

### Opportunistic infections

Although etanercept may have the lowest incidence of TB within the TNFi class, a recent study showed the risk was still significantly higher compared to rituximab with an adjusted HR of 4.63 (95% CI: 1.06, 20.2) [9]. However, CIs were wide because of low numbers of events. Also, rituximab is usually given as second-line treatment, and it may be expected that the risk with first biologic may be higher (having received a TNFi drug that has not lead to TB reactivation).

For non-TB opportunistic infections, the absolute risk reassuringly is low at approximately 1/1000 pyrs [9]. Thus far, there have been no significant differences in non-TB opportunistic infections between drugs, however, analysis of newer agents in the TNFi class or the non-TNFi biologic currently lack power to fully determine comparative risk of such rare events.

## CONCOMITANT THERAPY

### Conventional synthetic disease-modifying antirheumatic drugs and glucocorticoids

csDMARDs such as methotrexate are not associated with an increased infection risk in RA patients, without the use of glucocorticoids [21]. However, glucocorticoid use is likely to be one of the most important factors in terms of risk stratification of infection before starting and during biologic therapy. Dose, recency and duration of glucocorticoid prescription have been shown to be the most important factors when considering the risk of serious infection in RA patients [22]. For instance, a patient with a prescription of prednisolone of 5 mg for 3 months has a 30% increased serious infection risk but this goes up to 100% if used continuously for 3 years (in the absence of a co-prescribed biologic).

### Perioperative risk

The latest American College of Rheumatology/American Association of Hip and Knee Surgeons guidelines 2017 now recommend utilizing the dosing interval of biologics rather than their half-life in determining the withholding interval pre-surgery, whilst timing surgery at the nadir of the drug effect at the end of the dosing interval [23^▪▪^]. The evidence around the use of biologics in the perioperative period remains limited. In a US Medicare-based study, RA patients receiving infliximab within 4 weeks of elective hip or knee arthroplasty were not at a higher risk of postoperative infection within 30 days compared with patients withholding therapy for 8-12 weeks before surgery. More importantly, glucocorticoid use, especially more than 10 mg/day, was linked with an increased 30-day postoperative infection risk [OR 2.11 (95% CI 1.30–3.40)] and prosthetic joint infection risk within 1 year [HR 2.70 (95% CI 1.30–5.60)] [24^▪^]. Hence, tapering steroids as soon as possible, when disease activity is well controlled, is an essential strategy to reduce infections for all patients regardless of their infection risk otherwise.

### Opioids

Opioids are frequently administered in musculoskeletal conditions with 40% of RA patients using prescription opioids daily in a US study [25]. One emerging concern with opioids is the risk of infection. Certain opioids affect lymphocyte and phagocyte proliferation, reduce innate immune cell activity and inhibit cytokine expression and antibody production in animal studies [26]. A previous epidemiological study concluded an increased risk of serious infection in RA patients on opioids, using a self-controlled case series design to allow within-person comparisons when the patient was either on or off drug [27]. Risk of serious infection was higher with use of long-acting opioids, immunosuppressive opioids (codeine, morphine, transdermal fentanyl) and those with a daily morphine milligram equivalent (MME) dose of at least 60 mg. Most recently, opioids were found to be an independent risk factor of invasive pneumococcal disease, which include serious infection such as bacteraemia, meningitis and invasive pneumonia [28]. In that study, as well as the above risk factors, the risk of infection was increased with higher daily dosages [50–90 MME/day: OR, 1.71 (CI 1.22, 2.39]; ≥90 MME/day: OR, 1.75 (CI, 1.33,2.29)] [29^▪^]. Whether this risk increases further when combined with biologic therapies remains unknown. Therefore, although large biologic cohorts remain one of the best study designs to investigate risk of infection, one limitation among many is the lack of accurate exposure information on time-varying steroid and opioid use/dose that are likely to be co-prescribed in a large proportion of patients.

### Denosumab

Chronic uncontrolled inflammation in RA is a recognized risk factor for osteoporosis, as are glucocorticoids with dose, duration and recency associated with first osteoporotic fracture [30]. Given such risk of bone loss, patients are often co-prescribed therapies for osteoporosis. Denosumab is a fully human monoclonal antibody that binds to receptor activator of nuclear factor-κ-B ligand to inhibit osteoclast formation and bone resorption. Receptor activator of nuclear factor-κ-B ligand is also expressed on activated B and T lymphocytes and in lymph nodes; therefore, inhibition of this pathway may theoretically increase infection risk, especially in patients already on biologics.

There has been a concern about the added risk of infection among patients with RA receiving more than one biologic simultaneously and is therefore contraindicated [31]. However, analysis have not confirmed that this risk extends to the combination of biologic used for RA and denosumab. There is a low absolute risk of serious infection while on denosumab [32] and a recent small retrospective study reported a low risk of serious infection and opportunistic infection in patients receiving both a RA biologic and denosumab [33]. In RA patients treated with a biologic, the risk of serious infection with denosumab appears no higher than being on zoledronic acid [34]. Thus, the risk of infection with concomitant use of a biologic and denosumab with evidence to date appear reassuring.

### Treatment stratification for individual patients

As summarized above, there continues to be a wealth of new information generated assessing risk of infection on a population level, in different subgroups of patients and more recently between biologics, although the strength of association between drugs and infection risk remains debatable. Quantification of benefit and harms with accurate estimates of absolute and relative risk is ideally needed to inform decisions about the optimal therapeutic strategy. The reason for this is, in general, clinicians overestimate the benefits and underestimate harms of medications: indeed, a recent systematic review suggested these are only estimated accurately 11 and 13% of the time, respectively [35]. Current understanding of this balance and subsequent prescribing is often based on factors such as clinician intuition, cost of medications and generic guidelines.

### Risk prediction scores

Estimating the risk of infection before treatment and during the course of therapy would considerably aid in personalized decision-making. For instance, healthier patients may be channelled to restart biologics after serious infection [36], however, the decision to restart after serious infection maybe much more challenging in the absence of quantifiable risk in older multimorbid patients. A number of risk scores have been developed for this purpose in different populations, with similar variables of increasing age, glucocorticoid use, comorbidities such as chronic lung disease/renal disease and previous serious infection [37–39], however, none have been validated in external data sources (Table 1). More recently, age-adjusted comorbidity indices have been developed to predict the risk of serious infection in RA-treated certolizumab pegol patients using baseline characteristics [40]. The Rheumatoid Arthritis Observation of Biologic Therapy (RABBIT) risk score has an online calculator that can be a useful tool for stratifying risk and can include time-varying components such as glucocorticoid dose; however, a few limitations exist. The risk score has been replicated in a contemporary cohort within the same healthcare setting, but not in other international populations. It clearly states that it should not to be used as an indicator for the appropriateness of treatment decisions. A probability of serious infection over 1 year is generated; however, how this translates to absolute risk that is easily understandable for patients is unclear. Additionally, although these tools are clearly a huge advance in helping informed decisions, whether use improves patient outcomes long-term has not been assessed.

**Table 1:** Risk prediction scores for serious infection in rheumatoid arthritis patients

				Risk factors for SI included in the prediction model					
Author/Year	Data collection period	Study population	No. of SI events (sample size)	Demographics	Comorbidities	Disease activity/burden indicators	Treatment-related factors	Performance (discrimination/calibration)	Validation
Strangfeld 2011 (RABBIT risk score) [39]	2001–2006	RA enrolled in the German biologics register RABBIT	392 (5044)	Age >60 years	Chronic lung disease Chronic renal disease History of serious infections	Functional capacity (measured using Hannover Functional Status Questionnaire)	Glucocorticoid dose (7.5–14 mg or ≥15 mg/day) at baseline or follow-up High number of csDMARDs and bDMARD treatment failures (>5) at baseline	Similar expected and observed SI rates in deciles of risk scores using the Hosmer–Lemshow test (3 SIs per 100 patient-years for both in validation cohort)	1522 TNFi-treated patients in the same register (data collection period: 2009–2012) [41]
Crowson 2012 [37]	RA diagnosed between 1955–1994, followed up to 2000	US-based Minnesota residents with incident RA (not biologic treated)	491 over 10 years (584)	Age: 60–80 or ≥80 years (highest risk)	Number of comorbidities^a^ (≥1) History of SI in the last year (highest risk) or in last 2–3 years	Extra-articular manifestations of RA^b^ ESR >30 or >50 (highest risk)	Glucocorticoid use (>10 mg/day highest risk)	C statistic 0.81 (95% CI: 0.75–0.86) Calibration performed by comparing predicted and observed 1-year SI risk by deciles of predicted probability (similar)	410 RA patients from same cohort (with RA diagnosis 1995–2007)
Curtis 2012 [38]	2005–2010	Medicare and Medicaid patients (csDMARD and TNFi-treated patients)	1549 (14,693 government insured patients; 213 (8823 commercially insured patients)	Age ≥65 years (increasing for subsequent decades)	Diabetes (with or without complications) COPD Heart failure Malignancy Angina Peptic ulcer disease Hepatitis C Renal disease Any fracture Skin ulcers Previous SI	Long-term care Disabled Health services utilization (e.g. any cause hospitalization)	Glucocorticoids >7.5 mg/day Narcotics Antifungal, hypertension, antidepressant, NSAIDs, thiazides medications; intra-articular glucocorticoid injections, bisphosphonates	C-statistic for governmentally insured patient model was 0.71 (95% CI: 0.69–0.72) and for commercially insured patients: 0.78 (95% CI 0.75–0.80) Calibration performed by comparing predicted and observed 1-year SI risk by deciles of predicted probability (similar)	Validation cohort derived from 200 bootstrap samples (of equal size to the original data set)
Curtis 2017 [40]	2005–2006 (follow up to 2011 as part of OLE)	Pooled RA patients on certolizumab from RCTs RAPID1/2	40 (1224)	Age ≥70 years	Diabetes COPD Hyperlipidaemia Osteoporosis Depression	NA (baseline DAS28 and erosion scores not associated with SI in pooled cohort)	Systemic glucocorticoids at baseline (yes/no)	C-statistic was 0.85 (95% CI: 0.73–0.93) Predicted SI rates similar to observed rates, suggesting a well-calibrated model	Not validated

bDMARD, biologic disease-modifying antirheumatic drugs; csDMARDs, conventional synthetic disease-modifying antirheumatic drugs; COPD, chronic obstructive pulmonary disease; DAS28, disease activity score of 28 joints NA, not applicable; OLE, open-label extension; RA, rheumatoid arthritis; RABBIT, Rheumatoid Arthritis Observation of Biologic Therapy; RCT, randomized controlled trial; SI, serious infection; TNFi, tumour necrosis factor inhibitor.

^a^Any of the following: diabetes, chronic lung disease, alcoholism, coronary heart disease, heart failure and peripheral vascular disease.

^b^Amyloidosis, Felty's syndrome, rheumatoid vasculitis and rheumatoid lung disease.

A major limitation of using baseline characteristics to inform risk scores is that in the majority of patients most risk factors are non-modifiable. For instance, a 75-year-old man with a serious infection in the last 12 months, known chronic obstructive airways disease, on 15 mg of prednisolone starting etanercept 50 mg/week has a risk of 33.7% of serious infection during the next 12 months [42]. However, apart from the decision not to treat at all or reducing glucocorticoid dose, there are a few factors that either the clinician or patient can influence to attenuate risk. Thus far, there have been no implementable biomarkers that can either predict or help modify risk of serious infection. One possible pharmacological biomarker is biologic drug levels that have been previously shown to be associated with long-term treatment response and adherence to biologic treatments [43–45]. Recent emerging evidence from the United Kingdom suggests biologic drug levels could be associated with infection risk over 12 months [46]. Therefore, in the future guided by evidence, biologic therapeutic drug monitoring could be used to maintain therapeutic drug levels to preserve efficacy to the drug, help guide tapering [47], improve adherence strategies, whereas reducing infection risk in patients.

## CONCLUSION

The last decade has seen tremendous advances in our understanding of biologic safety and the majority of evidence has provided reassurance to patients and prescribers. In the last year, some advances have been made in our understanding of comparative safety, specific clinical scenarios such as perioperative risk, as well as consideration of potential effect modifiers such as opioids that may themselves increase infection risk. As we embark on a new era of precision medicine, it will become increasingly pertinent to include not only patient demographic factors, but also available biomarkers, lifestyle factors, concomitant treatments including analgesia, environmental factors, patient behaviours and preferences to fully personalize treatment for individuals. Strides toward this goal need to be underpinned by robust scientific evidence and likely incorporate a range of data sources and novel linkages to good quality drug exposure data. Although such an approach may appear ambitious, improved quantification of personalized risk and benefits would allow better-informed decisions, reduction of morbidity as well as reductions in direct and indirect costs associated with these drugs.

## Acknowledgements

M.J.'s work is supported by an NIHR academic clinical lectureship. We acknowledge the support of Arthritis Research UK Centre for Epidemiology (grant reference 21755), the Arthritis Research UK Centre for Genetics and Genomics (grant reference 21754) and the NIHR Manchester Biomedical Research Centre. The views expressed are those of the authors and not necessarily those of the NHS, the NIHR or the Department of Health.

### Financial support and sponsorship

None.

### Conflicts of interest

M.J. and A.B. declare no relevant COIs. K.H: Research grants from Pfizer, BMS and UCB; consultancy fees from Abbvie (all paid to host institution).

## REFERENCES AND RECOMMENDED READING

Papers of particular interest, published within the annual period of review, have been highlighted as:

▪ of special interest
▪▪ of outstanding interest
